# Tabes Mesenterica

**Published:** 1902-02-01

**Authors:** 


### TABES MESENTERICA.

The great question in regard to tabes mesenterica is as to its relation to milk, for while by some it is maintained that the administration of new milk is one of the most important steps in its prevention, by others it is held that milk (containing tubercle bacilli, of course,) is its chief cause. In an address recently delivered before the Wimbledon Medical Society Dr. Vivian Poore[1] examined this question very carefully. He said that although the number of deaths from phthisis and most tuberculous diseases had diminished during recent years, an exception had to be made in regard to deaths from tabes mesenterica, and it had been assumed by some that as children presumably drink more milk in the first year than in any subsequent year, it might be taken that the cause of the increased mortality from tabes mesenterica in the first year of life was the bacilli contained in the milk coming from cows with tuberculous udders. This, however, Dr. Poore thought was a very large assumption, and he read the matter in another light. In the ten years 1881-1890 there were 8,890,238 children born in England and Wales, and of these the great majority we may assume were milk drinkers. Dividing those milk-consuming babies into two classes, we find that whereas 30,194 died of tabes mesenterica, 8,861,144 did not die of that disease, so that the odds against a baby dying of tabes mesenterica during the period of greatest milk-consuming appear to be 228 to 1. Again, working out the figures in regard to infants dying under one year of age, he arrived at the conclusion that "the odds are seven to one against a new-born child dying in its first year, but if it does die the odds are 32 to 1 against its dying of tabes." If, then, in 100 milk-drinking children dying in their first year only three die of tabes, that does not, he said, give a high degree of probability that milk and tabes are cause and effect. Even taking all the tuberculous diseases together, including both tabes and phthisis, they at the outside caused only about one in 16 of the deaths of these milk-drinking babies, as against one in eight of all deaths of the entire population at all ages and from all diseases. Turning now from the statistical side of the question, he said that, before deciding that tabes mesenterica is a form of tuberculosis produced by milk-drinking, one ought to be able to demonstrate by post-mortem examination that so-called tabes is in all cases tuberculous. But this cannot be done. Tabes, indeed, is often a euphemism for "starvation" or for "congenital syphilis," and, although a convenient term, is one of low pathological value. Again, are the tuberculous mesenteric glands, even supposing them to exist, due to a primary inoculation of the alimentary system? Most pathologists assert that primary tuberculous disease of the alimentary canal is not at all common, and that it is the rule, rather than the exception, to find caseous bronchial glands or other evidence of tubercle in the body. If this is so, it is quite impossible to conclude that the condition of the mesenteric glands or the ulceration in the intestines is really primary. Dr. Poore then proceeded to show how weak is the chain of evidence in regard to the presence of the bacillus in the milk, contrasting the experience of Dr. Ballard in regard to milk typhoid, which was found "not amongst the very poor, but amongst the people who had money enough to buy lots of milk," with our common experience which points to the prevalence of tabes "among the very poor indeed," and he summed up by saying that the probability is that milk-drinking as a cause of tuberculous disease of the bowels is of no very great importance. To clinch the matter he quoted figures to show that "those who are habitually brought presumably into close contact with the bacillus tuberculosis, such as the physician who tends the patient, the clergyman who visits him and buries him, the dairyman who sells tuberculous milk and butter, the butcher who deals in the meat of tuberculous oxen, and the agriculturist who tends the tuberculous cows and spreads their tuberculous dung upon the soil, show no excessive tendency to phthisis;" a way of putting the case which, we may say, is striking enough, but hardly science. Lastly, what is our practice in the matter? Do we regard tabes in a child as due to milk or to want of milk? At University College Hospital they have a large Samaritan Fund, a good deal of which they spend in giving away new milk to children that want it. Are they giving them meat or are they giving them poison? The practical point is that they thrive upon it to a wonderful degree.

[1] Clinical Jour., Jan. 8.

